# Gingival Anaplastic Large-Cell Lymphoma Mimicking Hyperplastic Benignancy as the First Clinical Manifestation of AIDS: A Case Report and Review of the Literature

**DOI:** 10.1155/2013/852932

**Published:** 2013-06-13

**Authors:** Rafaela Elvira Rozza-de-Menezes, Stefânia Jeronimo Ferreira, Diogo Lenzi Capella, Stephan Schwartz, Ana Helena Willrich, Lúcia de Noronha, Aline Cristina Batista Rodrigues Johann, Paulo Henrique Couto Souza

**Affiliations:** ^1^Department of Stomatology, Faculty of Dentistry, School of Health and Biosciences, Pontifical Catholic University of Paraná, Imaculada Conceição, 1155 Prado Velho, 80-215-901 Curitiba, PR, Brazil; ^2^Departament of Pathology, Erasto Gaertner Hospital, Dr. Ovande do Amaral, 201-Jardim América, 81-520-060 Curitiba, PR, Brazil; ^3^Department of Pathology, School of Medicine, Pontfical Catholic University of Paraná, Imaculada Conceição, 1155 Prado Velho, 80-215-901 Curitiba, PR, Brazil; ^4^Department of Oral Pathology, Faculty of Dentistry, School of Health and Biosciences, Pontifical Catholic University of Paraná, Curitiba, Imaculada Conceição, 1155 Prado Velho, 80-215-901 Curitiba, PR, Brazil

## Abstract

This paper presents an unusual case of gingival ALCL, which mimicked a benign hyperplastic lesion that occurred in a 57-year-old white man representing the first clinical manifestation of acquired immunodeficiency syndrome (AIDS). The patient was referred to the Dental Clinic of PUCPR complaining of a lobulated nodule on the gingiva of his upper central incisors. The presence of advanced chronic periodontitis and dental plaque raised suspicion for a benignancy. An excisional biopsy was performed, and large pleomorphic cells with an abundant cytoplasm, sometimes containing prominent nucleoli and “Hallmark” cells, were observed through hematoxylin and eosin staining. The tumor cells showed strong CD30 expression, EMA, Ki-67, and LCA, and negative stain for p80^NPM/ALK^, CKAE1/AE3, CD20, CD3, CD56, and CD15. The final diagnosis was ALCL (ALK-negative). Further laboratory tests revealed positivity for human immunodeficiency virus (HIV). The patient was submitted to chemotherapy, but four months after diagnosis, the patient died due to pneumonia and respiratory failure. Oral anaplastic large-cell lymphoma (ALCL) is a rare disorder. Only 5 cases involving the gingiva have been reported, and to our knowledge, this is the first case reported of the ALCL, which mimicked a hyperplastic benignancy as the first clinical manifestation of AIDS.

## 1. Introduction

Malignant lymphomas (MLs) are a large group of neoplasms affecting the lymphoid system and are subdivided into two main groups: Hodgkin's and non-Hodgkin's lymphomas (NHLs) [1]. ML is considered an opportunistic neoplasm because it frequently affects immunocompromised patients. The prevalence of ML has increased considerably with improved acquired immune deficiency syndrome (AIDS) survivorship [2, 3]. NHLs appear in 3% of HIV-seropositive patients [4]. The NHLs may be present outside the lymphoid system, in which case they are called extranodal [5]. This type of lymphoma is located at an extranodal site in 40% of cases [5] and its extranodal presentation occurs in almost 75% of human-immunodeficiency-virus- (HIV-) seropositive patients [2]. Furthermore, only 2% to 3% of these extranodal cases occur in the oral cavity [6]. 

In the group of NHLs, mature T cell and natural killer (NK) cell neoplasms make up only 12% of NHL cases worldwide [1]. Within this heterogeneous category, anaplastic large-cell lymphoma (ALCL) is found [7, 8] and is listed in the Revised European-American Classification of Lymphoid Neoplasms (REAL) as an independent entity [9]. A couple of ALCL cases have been reported, although some were the B-cell phenotype now classified as an anaplastic type of diffuse large B-cell lymphoma [10]; therefore, these cases were excluded from the present review. ALCL is characterized by proliferation of the anaplastic large lymphoid cells with abundant cytoplasm and strong expression of CD30 antigen; however, its final diagnosis is a challenge for oral pathologists [8, 11–17]. Two distinct clinical entities of ALCL are recognized by the World Health Organization (WHO) lymphoma classification: cutaneous and systemic lymphomas [1, 5]. The cutaneous ALCLs are characterized by indolent growth and may show spontaneous remission and good prognosis [18, 19]. The systemic ALCLs can be ALK-positive or -negative [18]. The ALK-positive ALCLs usually occur in male pediatric patients and show a good prognosis taking into account their affective response to chemotherapy, whereas the ALK-negative ALCLs occur in elderly patients and present a poor prognosis [18, 20]. 

ALCL especially affects the skin of the body [6]. This T-type lymphoma in the oral cavity is an extremely rare disorder characterizing 1% of lymphomas overall [19]. From the scientific literature, we found around 15 cases of ALCL in the oral cavity [2, 6, 8, 11–17, 21, 22]. Only 5 cases of ALCL located in gingiva have been reported in the literature [12, 17, 22], and our case is the fourth reported occurrence of ALCL in the oral cavity of a HIV-seropositive patient [2, 11]. Besides, this paper is the first to present an unusual case of oral ALK-negative ALCL that mimicked a benign hyperplastic lesion as a first clinical manifestation of AIDS.

## 2. Case Report

**Figure 1:**
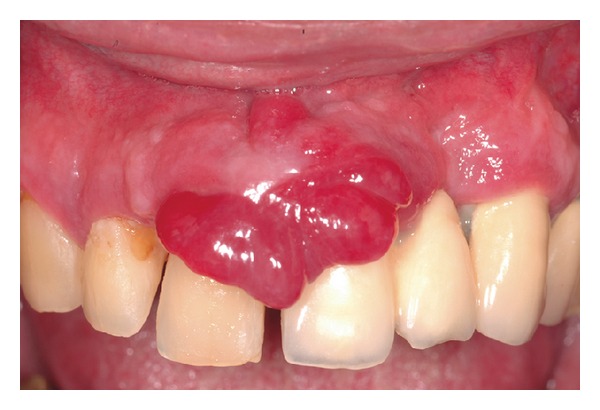
Clinical presentation: a 2,5 cm red nodule, pedunculated, located on the gingival between upper central incisors.

**Figure 2:**
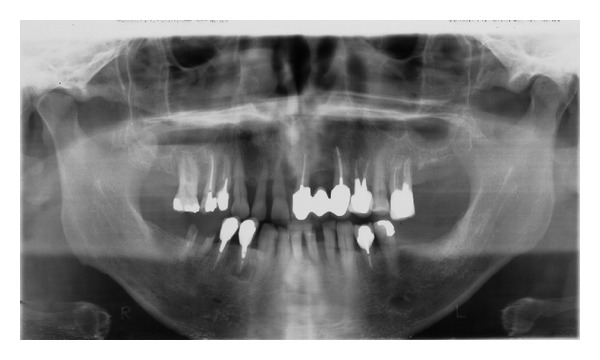
Radiographic image: panoramic radiography showing the extensive bone resorption in the anterior area of the maxilla compatible with advanced periodontal disease.

A 57-year-old white man was referred to the Dental Clinic of PUCPR complaining of an asymptomatic hyperplastic lesion on the gingiva of his upper central incisors, which had grown significantly in the last two months. The patient was a nonsmoker and his medical history did not reveal other relevant information. The extraoral examination showed a small swelling of the upper lip and absence of cervical lymphadenopathy. The intraoral examination showed advanced chronic periodontitis and the presence of a lobulated reddish nodule, pedunculated, measuring around 2.5 cm on the gingival area between both upper central incisors which had mobility and accumulation of dental plaque (Figure 1). The lesion did not have an ulcerated surface, and palpation revealed a soft consistency with bleeding on the surface. Panoramic radiography showed a significant resorption of bone mainly in the anterior area of the maxillary, compatible with advanced chronic periodontitis (Figure 2).

Taking into account that the first impression of the lesion revealed clinical characteristics of a benignancy, as the pedunculated nodule, circumscribed, freely moveable, the presence of dental plaque, and advanced chronic periodontitis, we initially suspected that it could be related to a reactive hyperplastic lesion. These lesions include pyogenic granuloma, focal fibrous hyperplasia, peripheral giant cell granuloma, and peripheral ossifying fibroma. Therefore, considering these main hypotheses, an excisional biopsy of the nodule was performed under local anesthesia. Furthermore, the patient was referred for periodontal treatment.

**Figure 3:**
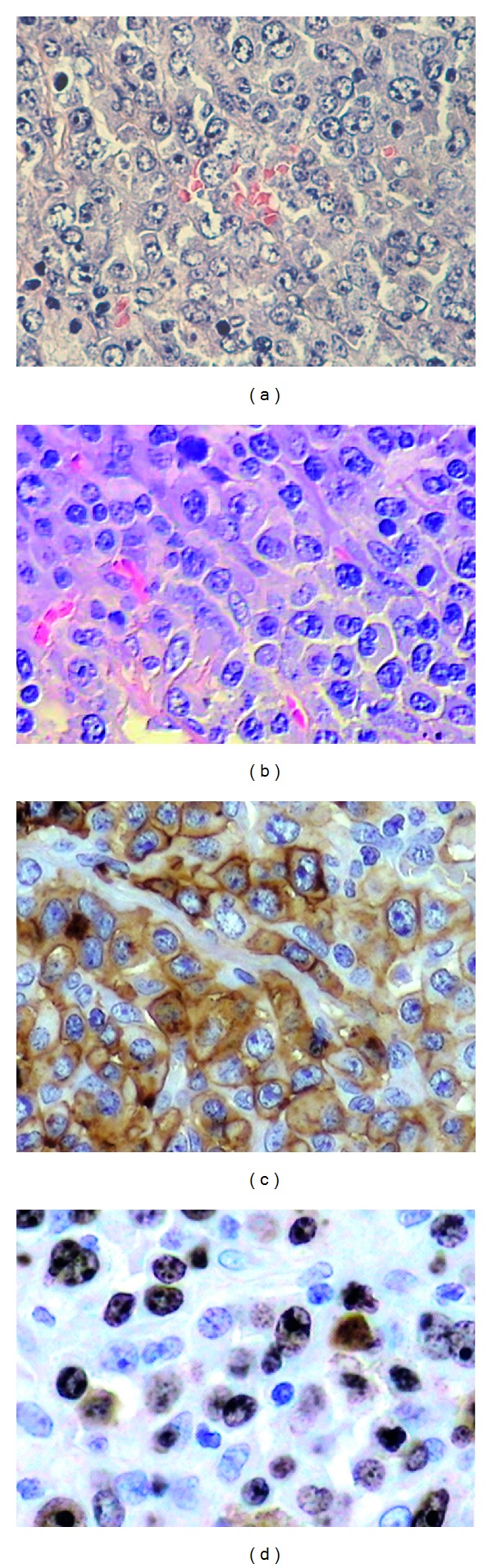
Histologic features: (a) diffuse proliferation of lymphoid tumor cells (hematoxylin-eosin stain (HE), magnification ×200) was observed. (b) Large pleomorphic cells with an abundant cytoplasm, sometimes containing prominent nucleoli, were verified. “Hallmark” cells with eccentric, horse-shoe, or kidney-shaped nuclei were also identified (HE, magnification ×400). Immunohistochemical findings: sections showing tumor cells are positive for (c) CD30 with a membrane and Golgi distribution (streptavidin-biotin, ×400 magnification) and (d) Ki-67 (streptavidin-biotin, ×400 magnification).

Hematoxylin and eosin-stained sections showed a lesion characterized by a diffuse proliferation of lymphoid tumor cells underneath an epithelium (Figure 3(a)). The slices also showed large pleomorphic cells with an abundant cytoplasm, sometimes containing prominent nucleoli. “Hallmark” cells with eccentric, horse-shoe, or kidney-shaped nuclei were also observed (Figure 3(b)). The tumor cells frequently demonstrated mitotic figures. Immunohistochemical analysis using a streptavidin-biotin protocol [14] was performed with the antibodies: CKAE1/AE3, CD20, CD3, CD30, Ki-67, epithelial membrane antigen (EMA), CD56, leukocyte common antigen (LCA), p80^NPM/ALK^, and CD15. The tumor cells showed strong CD30 expression with a membrane and Golgi distribution in the majority of tumor cells (Figure 3(c)). Ki-67 positive stain was observed in 90% of tumor cells (Figure 3(d)). Positive stain in tumor cells was also identified for EMA and LCA. Negative stain was identified for p80^NPM/ALK^, CKAE1/AE3, CD56, and CD15. The tumor was a null-cell type (negatively stained for CD20 and CD3). Based on clinical, histological, and immunohistochemical findings, the final diagnosis was extranodal ALK-negative ALCL.

The patient was referred to a specialized oncology hospital where further laboratory and imaging tests showed positivity for HIV, negativity for both hepatitis B and C, normal full blood count proportions of white and red blood cells and platelets, and absence of lymphadenopathies and metastasis. The following chemotherapy protocol consisted of six cycles of CHOP (cyclophosphamide 750 mg/m^2^, doxorubicin 50 mg/m^2^, vincristine 2 mg, and oral prednisone 100 mg) for five days [23]. However, four months after diagnosis, the patient died due to pneumonia and respiratory failure.

## 3. Discussion

This case report presents a rare case of ALCL in an extranodal site of a Brazilian male HIV-seropositive patient. Since ALCL was first described as a clinical entity by Stein et al. (1985) [20], this lesion was classified as Ki-1+ ALCL and currently as Ki-1 (CD30) [17], which corresponds to a cohesive proliferation of large pleomorphic cells that express CD30. The ALCL is subdivided according to the immunohistochemical features in precursor T cells or null cells, in 60% and 25% of the cases, respectively [2]. Therefore, immunohistochemistry is imperative for the correct diagnosis of ALCL considering that the cells are positive for CD30 antigen and several other markers such as EMA that is present in slightly more than 50% of cases [2]. LCA is usually positive but may be used negative in 30% to 40% of cases [2]. In most cases, other markers such as Ki-67, CD3, and CD45RO (T-cell phenotype), TIA-1 (T-cell intracytoplasmic antigen-1), perforin, granzyme B, and p80^NPM/ALK^ will be positive [6, 17]. 

The clinicopathologic features of ALK-carrying ALCLs were first investigated by Shiota and Mori [24]. The authors found that ALK-positive is a distinctive entity clinically and pathogenetically and should be differentiated from ALK-negative. Indeed, based on the most recent findings, the WHO classification currently considers two ALCL types, negative and ALK-positive [25, 26]. The tumors that show a positive reaction for ALK have greater cell proliferation and can show a relatively better prognosis [19]. Recently, studies have proposed that proteins such as aberrant fusion protein (NPM-ALK), JAK-STAT, and PI3K/STAT may be a potential target in ALK-positive lymphomas [26–28]. The findings about specific antigens also suggest that ALK represents an ideal tumor antigen for vaccination-based therapies of ALCL and other ALK-positive tumors [29]. On the other hand, the cases of ALK-negative ALCL show an inaccurate behavior with a relatively unfavorable prognosis [18]. To make the diagnosis of ALK-negative ALCL, there must be a large cell predominant population with abundant cytoplasm and pleomorphic, embryo or hallmark nuclei or wreath-like giant cells, and strong CD30 expression with a membrane and Golgi distribution in virtually every cell with a highly anaplastic T-cell phenotype [17, 19]. As the current case did not express ALK, it was classified as an ALK-negative ALCL. In addition, the tumor did not show expression of NK or T-cell markers; therefore, it was classified as a null cell lymphoma. ALK-negative ALCL normally occurs in adults (40–65 years) with a modest male prevalence [3], as seen in our patient, a 57-year-old man. 

Based on cases found in the review of the literature (Table 1), there is a slight male prediction (female : male, 1 : 1.5), and the patients presented at a mean age of 53.4 years old (standard deviation, 22.4). Most of the tumors were located in the gingiva (5 cases), followed by the palate (3 cases), tongue (2 cases), retromolar trigone (2 cases), lip (2 cases), floor of the mouth (1 case), and buccal mucosa (1 case). The main radiographic findings were bone resorption [11, 12, 17], and the main diagnostic hypothesis more frequent is nonspecific infection (4 cases) [2, 13, 16]. To the best of our knowledge, the present case reports the first gingival ALCL, which mimicked a benign hyperplastic lesion as the first clinical manifestation of AIDS. Positivity to HIV was reported in 3 cases [2, 11]. About 14% of the ALCL cases were ALK-negative [17]; our case is the fourth case reported [17, 22]. This highlights that studies about ALK protein were developed in the 1990s. Only in 2002 [16], p80^NPM/ALK^ was first included in the immunohistochemical studies involving oral ALCLs. Currently, there is only one case reported that showed ALK expression in ALCL involving oral cavity [16].

Interestingly, cases of ALCL present in the gingiva also suggested that the majority of clinical aspects mimicked gingivitis or advanced chronic periodontitis with ulcers, bleeding, and swelling. All clinical aspects of ALCLs in oral cavity were associated with extensive bone resorption, which made the clinical diagnosis of ALCL more difficult. Clinical cases have reported other entities of oral lymphomas that mimicked a benignancy in HIV-seropositive patients [30]. We found only 15 cases of oral ALCL reported in the English literature (Table 1). The current case showed an unusual appearance of a reactive hyperplastic lesion associated with advanced chronic periodontitis. Indeed, this fact emphasizes the need for a thorough clinical history, HIV investigation, and also high index suspicion in both dentists and pathologists. 

Oral lymphomas also occur more frequently in patients with HIV infection [31] as well as those iatrogenically immunosuppressed [31, 32]. Mechanisms other than the loss of immune surveillance appear to be involved with HIV-associated malignances [11]. In our case, the oral lymphoma with distinctive clinical appearance and associated with periodontitis occurred in an HIV-seropositive patient and was the first manifestation of AIDS. Some studies have also suggested that HIV is a risk factor for the development of ALCL, but this hypothesis is not clear yet. Similarly, the relative risk for the development of an NHL in HIV patients is approximately 3%, making the prognosis worse [2, 4]. A few cases of oral ALCL in patients with HIV infection have been reported in the literature [2, 11]. It is important to point out that ALCL prognosis is usually associated with immune status, and the death of the patient in this case due to the presence of pneumonia and respiratory failure was directly related to his poor immune systemic condition [20]. 

ALCL is normally treated by intensive chemotherapy with CHOP (cyclophosphamide 750 mg/m^2^, doxorubicin 50 mg/m^2^, vincristine 2 mg, and oral prednisone 100 mg from the first to the fifth day of chemotherapy) [23]. This CHOP regimen was administered every 3 weeks for a total of six cycles. Several studies demonstrate the high rate of complete remission (70%–95%) and 70%–85% long-term overall survival [23]. However, unfortunately, in the current case, the patient died.

## 4. Summary

In our case, it is important to highlight that the rapid growth of the lesion in the last two months reported by the patient and the presence of dental plaque and advanced chronic periodontitis indicated a reactive process in the gingiva. For this reason, the first decision to make an excisional biopsy of the lesion was kept. It is also important to stress that, if it was known that the patient was HIV-positive, our conduct would be completely different. Unfortunately, the final diagnosis was a malignant lesion.

We presented an unusual case of ALK-negative ALCL on the gingiva associated with advanced chronic periodontitis that mimicked some clinical features of a hyperplastic gingival benignancy, which manifested in a 57-year-old HIV-seropositive man. ALCL is a systemic malignant disease characterized by the extranodal type which rarely occurs in the oral cavity as the first manifestation of acquired immunodeficiency syndrome. Thus, we emphasize the importance of an accurate clinical, including HIV investigation, histological, and immunohistochemical analysis of oral lesions to establish the correct diagnosis of those lesions which in daily practice are considered benign.

## Figures and Tables

**Table 1:** Clinical, radiological, and ALK-profile features of oral anaplastic large-cell lymphomas.

Age	Gender	Oral area	Clinical findings	Radiographic findings	HIV	Diagnostic hypothesis	ALK	References
ND	ND	ND	ND	ND	ND	ND	ND	Takahashi et al. [8]
36	M	Retromolar trigone	Mass with ulcerated surface	Diffuse radiolucency with poorly margins	+	Nonspecific infection	ND	Hicks et al. [2]
42	M	Retromolar trigone	Enlargement surrounding a crateriform ulcer	Not relevant	+	Pericoronitis or nonspecific infection	ND	Hicks et al. [2]
12	F	Gingiva	Scaly, swollen, and bleeding gingival surface	Bone resorption causing teeth displacement	+	Gingivitis	ND	Willard et al. [11]
12	M	Hard palate	Mass involving the nasal cartilage and floor of the orbit	ND	ND	ND	ND	Papadimitriou et al. [21]
75	F	Upper gingiva	Periodontitis (deep pockets) and redness of the gingiva	Bone resorption adjacent to teeth 11, 12, and 13	ND	Periodontitis	ND	Rosenberg et al. [12]
61	M	Lower gingiva	Well-delimited ulceration	ND	ND	Eosinophilic granuloma	ND	Rosenberg et al. [12]
48	M	Upper lip	Nonfluctuant, firm, and swelling lesion	ND	ND	Nonspecific infection	ND	Chandu et al. [13]
76	F	Upper lip	Well-delimited ulceration	ND	ND	ND	ND	Chim et al. [14]
65	F	Hard palate, buccal mucosa, and floor of the mouth	Well-delimited ulceration	ND	−	ALCL	ND	Born et al. [15]
77	M	Left side of soft palate	Mass with ulcerated surface	ND	−	Lymphoma, salivary gland neoplasm, or necrotizing sialometaplasia	ND	Savarrio et al. [6]
77	M	Tongue	Nodule with ulcerated surface	A low density mass	ND	Nonspecific infection	+	Notani et al. [16]
76	F	Upper and lower gingiva	Swollen gingival	Bone resorption	ND	Chronic marginal periodontitis	−	Matsumoto et al. [17]
34	F	Upper gingiva	White/gray ulcerated mass with erythematous border	ND	ND	Lymphomatoid papulosis, fungal infection, or traumatic ulcer	−	Grandhi et al. [22]
53	M	Tongue	Mass with ulcerated surface	ND	ND	ND	−	Grandhi et al. [22]
57	M	Gingiva in maxillary incisor area	A lobulated reddish nodule, bleedin,g and pedunculated	Bone resorption	+	Reactive hyperplasic lesion^a^	−	Present case

ND: not described, F: female; M: male; +: positive; −: negative; ^a^includes pyogenic granuloma, focal fibrous hyperplasia, peripheral giant cell granuloma, and peripheral ossifying fibroma.
